# Integrated Hepatitis C Care for People Who Inject Drugs (Heplink): Protocol for a Feasibility Study in Primary Care

**DOI:** 10.2196/resprot.9043

**Published:** 2018-06-04

**Authors:** Geoff McCombe, Davina Swan, Eileen O’Connor, Gordana Avramovic, Peter Vickerman, Zoe Ward, Julian Surey, Juan Macías, John S Lambert, Walter Cullen

**Affiliations:** ^1^ School of Medicine University College Dublin Dublin Ireland; ^2^ Centre for Research in Infectious Diseases Mater Misericordiae University Hospital Dublin Ireland; ^3^ School of Social and Community Medicine University of Bristol Bristol United Kingdom; ^4^ Institute of Global Health University College London London United Kingdom; ^5^ Unidad de Enfermedades Infecciosas y Microbiología Hospital Universitario de Valme Seville Spain

**Keywords:** hepatitis C, primary health care, general practice, opiate substitution treatment

## Abstract

**Background:**

Hepatitis C virus (HCV) infection is a major cause of chronic liver disease and death. Drug use remains the significant cause of new infections in the European Union, with estimates of HCV antibody prevalence among people who inject drugs ranging from 5% to 90% in 29 European countries. In Ireland and the European Union, primary care is a key area to focus efforts to enhance HCV diagnosis and treatment among people who inject drugs.

**Objective:**

The Heplink study aims to improve HCV care outcomes among opiate substitution therapy (OST) patients in general practice by developing an integrated model of HCV care and evaluating its feasibility, acceptability, and likely efficacy.

**Methods:**

The integrated model of care comprises education of community practitioners, outreach of an HCV-trained nurse into general practitioner (GP) practices, and enhanced access of patients to community-based evaluation of their HCV disease (including a novel approach to diagnosis, that is, Echosens FibroScan Mini 430). A total of 24 OST-prescribing GP practices were recruited from the professional networks and databases of members of the research consortium. Patients were eligible if they are aged ≥18 years, on OST, and attend the practice for any reason during the recruitment period. Baseline data on HCV care processes and outcomes were extracted from the clinical records of participating patients.

**Results:**

This study is ongoing and has the potential to make an important impact on patient care and provide high-quality evidence to help GPs make important decisions on HCV testing and onward referral.

**Conclusions:**

A substantial proportion of HCV-positive patients on OST in general practice are not engaged with specialist hospital services but qualify for direct-acting antiviral drugs treatment. The Heplink model has the potential to reduce HCV-related morbidity and mortality.

**Registered Report Identifier:**

RR1-10.2196/9043

## Introduction

### Hepatitis C as a Global Challenge

With approximately 71 million people affected worldwide and 399,000 related deaths, chronic hepatitis C virus (HCV) infection is associated with considerable morbidity, including cirrhosis and hepatocellular carcinoma [1]. Injecting drug use remains the significant cause of new infections in the European Union, with estimates of HCV antibody prevalence among people who inject drugs (PWID) ranging from 5% to 90% in 29 European countries [2]. With successful treatment and viral eradication, recent data have demonstrated decreases in all-cause mortality, mortality due to cirrhosis, and the incidence of hepatocellular carcinoma [3-5].

Recently developed HCV direct-acting antiviral drugs are well tolerated and delivered for shorter courses (8-12 weeks), with trials reporting more than 90% cure rates among PWID [6]. Despite these highly effective and simplified therapeutic regimens, many people at risk are unaware of their infection, and obstacles limit access to HCV care, resulting in many patients not being treated [7].

Notwithstanding these challenges, the World Health Organization has set a goal of eliminating viral hepatitis as a major public health threat by 2030, reducing new chronic infections by 90% and reducing mortality by 65% [8]. In most countries, the scale-up of HCV treatment for PWID, along with needle and syringe programs and opiate substitution therapy (OST), will be key to these targets being achieved.

### Enhancing Access to Treatment

Reaching and engaging PWID with HCV are key challenges, given the prohibition surrounding illicit and injecting drug use and the resultant stigma and discrimination concerns and possible mistrust of health services [9]. Community and peer involvement is likely to be critical to the success of HCV prevention programs for PWID. It has also been hypothesized by some clinicians and researchers that successful treatment of HCV in PWID can improve recovery and engagement with addiction treatment [10].

Blood tests and liver biopsy were historically the standard approach to assess the need for treatment in those with chronic HCV infection. However, many patients have defaulted from HCV care because of perceived dangers associated with liver biopsy [11]. More recently, alternative diagnostic modalities including liver stiffness measurement by transient elastography (Echosens FibroScan Mini 430) have resulted in noninvasive approaches to stage fibrosis. Several European studies have recently reported on the feasibility of fibroscanning as a screening tool for drug users, with high rates of acceptance and uptake within various treatment and street outreach settings [12,13].The mobility of this equipment means it can be transported to community sites to access patients, and for PWID, this procedure can help assess disease severity, enhance HCV assessment, reduce patient identified barriers, target therapy, and enable the triage of patients for more immediate care [14-16].

### The Role of Primary and Community-Based Care

In Ireland and the European Union, primary care is increasingly providing long-term care for PWID. Although screening and identification practices are inconsistent, 62% to 81% of this population are infected with HCV [17,18]. Furthermore, research indicates 35% of patients attending general practitioners (GPs) for OST in Ireland also had problem alcohol use (PAU) [19], and a subsequent qualitative study highlighted the need for primary care to address this problem through screening and brief intervention [20]. The study recommended initial screening for PAU by the patient’s GP using the Alcohol Use Disorders Identification Test (AUDIT) screening tool [21] and providing feedback on results in all cases with positive findings through a brief intervention involving advice on minimizing alcohol harm, encouraging a reduction in alcohol consumption, and initiating referral to specialists when needed. Thus, primary care is a key area to focus efforts to enhance HCV diagnosis and treatment among PWID. However, a number of challenges exist in this regard, including for PWID: lack of awareness, fear of side effects for HCV drugs, possible poor adherence, and comorbid conditions; and for health care providers, limited knowledge and communication difficulties may be problematic [16,22,23]. These challenges can be addressed through education, audit and feedback, and liaison nurse support [24,25]. Research has demonstrated that if adequately supported, primary care and specifically general practice could adequately screen for HCV [26]. Research indicates that offering primary care practice staff training on the epidemiology, diagnosis, and management of HCV infection, so increasing testing of HCV was cost-effective [27]. Furthermore, testing PWID in primary care may also identify patients who have been diagnosed previously but have not been referred to, or failed to attend specialist care, offering the opportunity to review and follow-up such referrals.

### Aims

This Heplink study forms part of a European Union–funded program of research (HepCare Europe) which aims to optimize hepatitis C diagnosis; linkage between primary, secondary, and outreach community care; and access to treatment for at risk-populations in the European Union.

The Heplink study focuses specifically on developing complex interventions to enhance community-based HCV treatment and improve the HCV care pathway between primary care and secondary care. As such, Heplink is a prospective, nonrandomized pre-post intervention feasibility study in primary care to identify and invite patients on OST to undergo testing, with referral to secondary care, where appropriate. We will evaluate the feasibility, acceptability, and likely efficacy of this approach quantitatively, qualitatively, and through assessing its cost-effectiveness. The specific objectives of the study are to develop and implement an integrated model of HCV care and evaluate its feasibility, acceptability, likely efficacy, and cost-effectiveness in practice.

## Methods

### Study Design and Setting

This is a prospective, nonrandomized, pre-post intervention feasibility study to be conducted in OST-prescribing general practices from three sites across the Hepcare Europe consortium (Dublin, London, and Seville). Practices will be given written information on the study and asked to indicate their interest in participating. Our recent experience recruiting general practices [28,29] has taught us the importance of a researcher promptly following up an expression of interest from a GP to fully explain the study and highlight what will be required from the GP in terms of time and commitment to the study. As such, practices who express an interest in participating will be contacted by a member of the research team to explain the aims of the study and what is required.

### Study Population

A sample of 24 OST-prescribing GP practices will be recruited from the professional networks of members of the research consortium.

Practices will be eligible to participate if they are registered to prescribe methadone and have at least 10 patients currently receiving OST; agree to participate in practice audit; and (3) agree to facilitate nurse liaison.

Patients (n=240) will be eligible to participate if they are on OST (ie, methadone); are aged at least 18 years; and attend the practice for any reason during the recruitment period.

### Approach to Sampling and Recruitment

A standardized nonprobability sampling framework will be used to identify 10 consecutive patients from each practice or center to participate in the study. On the basis of the recommendations for good practice in feasibility studies [30] and our previous work conducting feasibility studies with PWID [29,31], we estimate that 240 patients (attending 24 general practices) will be adequate to calculate the actual recruitment and retention rates (ie, feasibility) and provide data on acceptability of study processes and outcome measures, which will inform a future definitive trial. This approach to recruitment reflects the challenges involved in recruiting this population group for projects. Patients who consult a doctor or nurse in participating practices or centers and who are eligible for the study will be given written information and a verbal explanation of the study, including the study purpose, procedures, and how findings will be utilized. Those who are interested in participating will be asked to sign a consent form, which will be witnessed by the doctor or nurse or the researcher. Although the initial approach to participate will be from a health care professional, recruitment will be facilitated by a member of the research team being “on site” to support the practice during the recruitment phase and answer any questions potential participants may have. The recruitment phase will involve each participating practice engaging in an intensive, 4-week period of patient recruitment, an approach we found most effective in previous work with this population [28,29]. During this 4-week period, the member of the research team will aim to (1) obtain contact details for and informed consent from eligible patients, (2) review the clinical records of patients who consent to participate in the study, and (3) collect baseline data, including patient demographics and current care process and outcome measures from clinical records.

### Intervention

**Figure 1:**
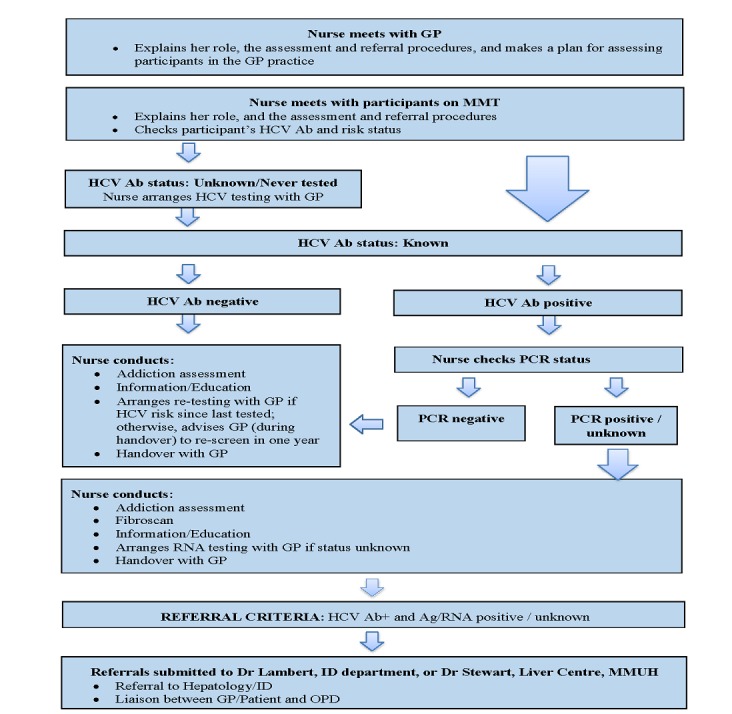
Flowchart of the nurse-led intervention. GP: general practitioner; HCV Ab: HCV antibody; HCV: hepatitis C virus; ID: infectious diseases; MMUH: Mater Misericordiae University Hospital; PCR: polymerase chain reaction; OPD: Outpatient Department; OST: opiate substitution therapy; RNA: Ribonucleic Acid; Ag/RNA: Antigen/Ribonucleic Acid.

Informed by the UK Medical Research Council “Framework for design and evaluation of complex interventions to improve health” [32], the aim of the intervention is to enhance identification and linkage to HCV care and treatment among patients attending primary care for OST and includes the following (see Figure 1):

- Outreach of an HCV-trained liaison nurse into GP practices
- In-practice education for GPs and practice staff regarding developments in diagnosis and treatment of HCV
- Enhanced access of patients to community-based evaluation of HCV disease, including a novel approach to diagnosis, that is, transient elastography (Echosens FibroScan Mini 430)
- Researcher-facilitated practice audit of HCV care processes and feedback to GP

### Data Collection

Data on patient demographics and HCV care processes/outcomes will be extracted from the patient’s clinical electronic or paper medical record and also from patient self-reported data collected through the HCV liaison nurse assessment to include the following:

- Prior HCV testing
- HCV status—antibody and Ribonucleic Acid (RNA) or antigen
- Referral and attendance for HCV care at a hospital’s Department of Hepatology or Infectious Diseases
- HCV assessment and treatment and sustained virological response (SVR)
- Alcohol screening and brief intervention using a validated alcohol screening instrument (eg, AUDIT)
- Other blood borne virus (BBV): HIV and hepatitis B virus (HBV)
- BBV testing/vaccination: liaison nurse will assess if the patient has been tested for HCV, HIV, and whether HBV immunization is required
- Chronic illness
- Mortality

The HCV liaison nurse will meet and assess the patient, addressing any questions they may have regarding their HCV status. The liaison nurse will also gather data on the following variables:

- HCV risk factors (eg, incarcerated, tattoos, piercings, blood transfusion, sexually transmitted infection test)
- OST treatment
- Alcohol use assessed using the AUDIT questionnaire
- HCV testing, assessment, and treatment history
- Other BBV testing and HBV and hepatitis A virus vaccination history
- Quality of life (EQ5D-3L)—collected by HCV liaison nurse just before their assessment and intervention where necessary with each patient
- HCV-antibody positive and Ribonucleic Acid/Antigen (RNA/Ag) status (positive or unknown)
- Echosens FibroScan Mini 430 data (the nurse will have received Echosens FibroScan Mini 430 training)

After the assessment, the liaison nurse will provide the patient with information/education regarding HCV, including how it is transmitted and how to prevent transmission. Harm reduction strategies will also be addressed if required. A handover of the nurse’s assessment will be given to the GP. HCV Ab+ patients will be referred to specialist services if they are Ag+, RNA+, or if Ag and RNA status are unknown. This process will be carried out by the GP with the assistance of the HCV liaison nurse. Referrals are submitted to the hospital’s Hepatology or Infectious Diseases department.

### Qualitative Data Collection

Semistructured interviews will be conducted by a member of the research team with health care professionals (n=12) and patients (n=12; or until analytical saturation is reached) at follow-up, purposively sampled from recruited practices and patients. Interviews will be guided by an interview schedule informed by our previous feasibility study in Irish primary care [33], including participants’ lived experiences of HCV including barriers and facilitators to treatment, as well as questions exploring participants’ experiences and acceptability of the study intervention. With purposive sampling, the researcher samples particular settings, persons, or events deliberately selected for the important information they can provide that cannot be acquired as well from other choices [34]. In line with the simultaneous analysis and collection of data that is an integral part of qualitative analysis, after the first 3 health care professional and first 3 patient interviews, recruitment will be informed by themes identified in the data. Participants recruited for the qualitative data collection will be provided with an amended participant information leaflet outlining the purpose of this part of the study, and, if agreeable, asked to sign a separate consent form.

### Outcome Measures

To establish the feasibility, acceptability, and likely efficacy of this intervention in practice, the following outcome measures will be examined (see Table 1 for measures to establish feasibility, acceptability, and likely efficacy):

Primary outcomes include the following:

- Proportion of participants who have been screened for HCV
- Proportion of HCV antibody–positive patients commenced on/completed antiviral therapy/achieved SVR

Secondary outcomes include the following:

- Proportion of those screened who tested HCV antibody positive
- Proportion of HCV-positive patients who have been assessed by Echosens FibroScan Mini 430
- Proportion of HCV-positive patients who have been referred to specialist hepatology or infectious diseases service
- Proportion of HCV-positive patients who have attended specialist hepatology or infectious diseases service
- Proportion of HCV-positive patients received an alcohol screening brief intervention
- Proportion of participants tested for anti-HIV antibody, anti-HBc (hepatitis B core) antibody, or hepatitis B surface antigen (HBsAg)
- Proportion of participants immunized against hepatitis B/A virus
- Experience and evaluation of the intervention among key informants (GPs, nurses, and patients)
- Number of patients attending general practice for OST postintervention for follow-up testing
- Evaluate feasibility and possible efficacy of intervention by comparing pre-post intervention data
- Evaluate the cost-effectiveness of the intervention
- Compare GPs’ and practice nurses’ knowledge and attitudes and practice pre-post intervention

### Data Analysis

Quantitative data will be analyzed using SPSS v22. Questionnaires will be summarized using descriptive statistics, and a pre-post intervention analysis will be performed using Fisher exact test.

Semistructured interview transcripts will be checked for accuracy and then imported into NVivo10 qualitative data analysis software to aid management and analysis of data. Analysis will begin shortly after data collection starts and will be ongoing and iterative. Analysis will inform further data collection; for instance, analytic insights from data gathered in earlier interviews will help identify any changes that need to be made to the topic guide during later interviews. Thematic analysis [35] using a data-driven inductive approach will be used to scrutinize the data to identify and analyze patterns and themes of particular salience for participants and across the dataset. The first author (a social psychologist with extensive experience in using qualitative methodologies) will analyze and code the data. The data will also be independently analyzed by the second author who also has extensive expertise in qualitative research. Final themes will be agreed between the 2 authors, and the last author will audit the final analysis.

The goals of this study are to examine feasibility, acceptability, and likely efficacy of the intervention. Descriptive statistics will be analyzed with respect to key feasibility variables. The qualitative analysis of patients’ and health care professionals’ experiences of the intervention will be used to assess acceptability. Although the study is not designed to determine effectiveness, we will examine likely efficacy by comparing process and outcome measures, which will allow estimation of the intracluster correlation coefficient and thus inform the sample size of a possible future definitive trial.

**Table 1:** Measures to establish feasibility, acceptability, and likely efficacy.

Aim	Patient level	Practice level
Feasibility	Compare pre-post intervention data on patient demographics, hepatitis C virus (HCV) care processes and outcomes extracted from patients’ clinical electronic or paper medical records, and also from patient self-reported data collected through the HCV liaison nurse assessment; patient recruitment rates; patient retention level; and cost-effectiveness of the intervention	Compare pre-post intervention data on patient demographics, HCV care processes and outcomes extracted from patients’ clinical electronic or paper medical records, and also from patient self-reported data collected through the HCV liaison nurse assessment; practice recruitment rates; practice retention level; cost-effectiveness of the intervention
Acceptability	Patients’ experience of intervention evaluated by analysis of semistructured interviews with patients	GPs’^a^ and practice nurses’ experience of intervention evaluated by analysis of semistructured interviews with patients
Likely efficacy	Although not adequately powered to examine efficacy, possible efficacy will be assessed by proportion of patients who have been screened for HCV and alcohol use, proportion of patients who have been referred for treatment (HCV, alcohol), and cost-effectiveness of study	Although not adequately powered to examine efficacy, possible efficacy will be assessed by knowledge and attitudes and practice pre-post intervention and cost-effectiveness of study

^a^GP: general practitioner.

### Cost-Effectiveness

A cost-effectiveness analysis will be undertaken of the Heplink intervention. This analysis will be important to investigate whether this HCV case-finding intervention is a worthwhile investment.

Costs for the intervention will be collected (in Euros) from the provider’s perspective using an ingredients-based approach, including a time and motion study of the nurse liaison activities. Health-related quality of life information will be collected using the EQ5D-3L (to calculate QALYs) at the start of the intervention, as there is a paucity of information for this patient group, with further quality of life data coming from other ongoing studies by the team. Using collected cost data and outcome data from the intervention, the long-term health benefits and costs of the intervention compared with the current standard of care will be calculated using a transmission dynamic model of HCV infection and disease progression. Further data to parameterize the model (PWID epidemiological and behavioral data and HCV transmission and progression data) will come from the published and gray literature. The incremental cost-effectiveness ratio will be calculated in terms of incremental cost per QALY saved and compared with the willingness-to-pay threshold for each country (€45,000 per QALY for Ireland) to determine whether the intervention is cost-effective.

### Ethical Considerations

The study has been approved by the Mater Misericordiae University Hospital Research Ethics Committee (Ref: 1/378/1722) and the Research Ethics Committees of the collaborating institutions in London and Seville.

Ethical considerations and safeguards include the following:

- Informed consent and consenting capacity: all potential participants (GPs, patients) will be given written information on the study, the model of care being proposed, and asked to provide written consent that they are happy to participate and that nonparticipation will not compromise their usual care. Participation in the study will be on a voluntary basis. No inducements to participate will be offered.
- Confidentiality: Any data/personal details that could potentially reveal the identity of individuals will be removed. Only anonymized, deidentified information will leave the practice of origin. To allow follow-up, an alphanumeric code will be assigned to each participant’s data; a database will be maintained on a password-protected database at Mater Misericordiae University Hospital. The list will be kept separately from patient data but will indicate the medical record number of each participant and the alphanumeric code. All research data will be stored on a password-protected desktop computer at the host organization. Study participants will be invited to give permission to have their name, address, and contact details held by the research team to facilitate their receiving a synopsis of the study findings on publication and to be contacted for follow-up data collection. All data will be stored securely at the host institution.
- Clinical governance, do no harm: it is possible that participating in the study may raise health-related issues for participants and may identify a health issue that requires a clinical intervention. Therefore, all participants will be advised to speak with their doctor if participating in the study has raised any such issues. At the conclusion of the intervention, the HCV-trained liaison nurse will conduct a handover of clinical information to the GP, which includes recommendations for follow-up care.

## Results

The 3-year project is funded, from June 2016, by the European Union Third Health Programme (grant agreement number 709844) and Ireland’s Health Services Executive. Data collection will be completed by July 2018, and data analysis is currently ongoing, with the findings expected to be submitted for publication in late 2018. Baseline data results from the Dublin arm of the study were submitted for publication in March 2018. The study findings have the potential to make an important impact on patient care and provide high-quality evidence to help GPs make important decisions on HCV testing and onward referral.

## Discussion

### Strengths and Limitations

“Heplink” is the first study to examine the feasibility and acceptability of integrated HCV care among problem drug users attending primary care in Europe. It will provide key data to enhance scientific understanding of interventions that prevent risk behaviors, inform policy and service development, and contribute to health and social gain locally and internationally. This study has the potential to make an important impact on patient care and will provide high-quality evidence to help GPs make important decisions on HCV testing and onward referral. The intervention is scalable and, therefore, if found to be feasible, acceptable, and cost-effective, it can be readily implemented elsewhere and used to guide policy and service development internationally.

Possible limitations of the study include potential issues of bias and lack of generalizability that may arise from the recruitment process, resulting in the likelihood that GPs who are more motivated and interested in research and innovation will choose to participate. Although target patient sample of 240 patients is relatively small for a trial study, it will allow us to estimate the sample size required for a future definitive trial.

The project team involves academic, clinical, and policy experts responsible for planning and delivery of addiction care and primary care and international experts on optimum primary care delivery to at-risk populations and primary care alcohol treatment.

The proposed work builds on our previously conducted work that outlined the barriers and enablers to HCV screening and treatment in practice and examined the effectiveness of a nurse-led intervention to enhance HCV screening in primary care [36].

### Conclusions

At the end of this research, the feasibility of a clinical intervention, informed by international best practice and local barriers, will be evaluated among a high-risk population. This feasibility study will inform clinical practice by providing initial indications as to whether a nurse-led integrated model of HCV care is feasible, acceptable, and also effective among problem drug users attending primary care. It will also inform future research on the topic by providing key parameters for the design of a future cluster randomized controlled trial.

## Abbreviations

AUDIT: Alcohol Use Disorders Identification Test
BBV: blood borne virus
GP: general practitioner
HBV: hepatitis B virus
HCV: hepatitis C virus
OST: opiate substitution therapy
PAU: problem alcohol use
PWID: people who inject drugs
QALY: quality-adjusted life year
RNA: Ribonucleic Acid
SVR: sustained viral response

## References

[1] World Health Organization http://apps.who.int/iris/bitstream/handle/10665/255016/9789241565455-eng.pdf?sequence=1.

[2] Lazarus JV, Sperle I, Maticic M, Wiessing L (2014). A systematic review of Hepatitis C virus treatment uptake among people who inject drugs in the European Region. BMC Infect Dis.

[3] van der Meer AJ, Veldt BJ, Feld JJ, Wedemeyer H, Dufour JF, Lammert F, Duarte-Rojo A, Heathcote EJ, Manns MP, Kuske L, Zeuzem S, Hofmann WP, de Knegt RJ, Hansen BE, Janssen HL (2012). Association between sustained virological response and all-cause mortality among patients with chronic hepatitis C and advanced hepatic fibrosis. J Am Med Assoc.

[4] Simmons B, Saleem J, Heath K, Cooke G, Hill A (2015). Long-term treatment outcomes of patients infected with hepatitis C virus: a systematic review and meta-analysis of the survival benefit of achieving a sustained virological response. Clin Infect Dis.

[5] Backus LI, Boothroyd DB, Phillips BR, Belperio P, Halloran J, Mole LA (2011). A sustained virologic response reduces risk of all-cause mortality in patients with hepatitis C. Clin Gastroenterol Hepatol.

[6] Dore GJ, Altice F, Litwin AH, Dalgard O, Gane EJ, Shibolet O, Luetkemeyer A, Nahass R, Peng CY, Conway B, Grebely J, Howe AY, Gendrano IN, Chen E, Huang HC, Dutko FJ, Nickle DC, Nguyen BY, Wahl J, Barr E, Robertson MN, Platt HL, C-EDGE CO-STAR Study Group (2016). Elbasvir-Grazoprevir to treat hepatitis C virus infection in persons receiving opioid agonist therapy: a randomized trial. Ann Intern Med.

[7] Zeremski M, Dimova RB, Pillardy J, de Jong YP, Jacobson IM, Talal AH (2016). Fibrosis progression in patients with chronic hepatitis C virus infection. J Infect Dis.

[8] World Health Organization (2016). http://apps.who.int/iris/bitstream/handle/10665/206453/WHO_HIV_2016.04_eng.pdf?sequence=1.

[9] Page K, Morris MD, Hahn JA, Maher L, Prins M (2013). Injection drug use and hepatitis C virus infection in young adult injectors: using evidence to inform comprehensive prevention. Clin Infect Dis.

[10] Alavi M, Spelman T, Matthews GV, Haber PS, Day C, van Beek I, Walsh N, Yeung B, Bruneau J, Petoumenos K, Dolan K, Kaldor JM, Dore GJ, Hellard M, Grebely J, ATAHC Study Group (2015). Injecting risk behaviours following treatment for hepatitis C virus infection among people who inject drugs: the Australian Trial in Acute Hepatitis C. Int J Drug Policy.

[11] Akay S, Karasu Z, Noyan A, Pala S, Musoglu A, Ilter T, Batur Y (2007). Liver biopsy: is the pain for real or is it only the fear of it?. Dig Dis Sci.

[12] Arain A, De Sousa J, Corten K, Verrando R, Thijs H, Mathei C, Buntinx F, Robaeys G (2016). Pilot study: combining formal and peer education with FibroScan to increase HCV screening and treatment in persons who use drugs. J Subst Abuse Treat.

[13] Marshall AD, Micallef M, Erratt A, Telenta J, Treloar C, Everingham H, Jones SC, Bath N, How-Chow D, Byrne J, Harvey P, Dunlop A, Jauncey M, Read P, Collie T, Dore GJ, Grebely J (2015). Liver disease knowledge and acceptability of non-invasive liver fibrosis assessment among people who inject drugs in the drug and alcohol setting: The LiveRLife Study. Int J Drug Policy.

[14] Grebely J, Morris MD, Rice TM, Bruneau J, Cox AL, Kim AY, McGovern BH, Shoukry NH, Lauer G, Maher L, Lloyd AR, Hellard M, Prins M, Dore GJ, Page K, InC Study Group (2013). Cohort profile: the international collaboration of incident HIV and hepatitis C in injecting cohorts (InC3) study. Int J Epidemiol.

[15] Sharma P, Dhawan S, Bansal R, Tyagi P, Bansal N, Singla V, Kumar A, Matin A, Arora A (2014). Usefulness of transient elastography by FibroScan for the evaluation of liver fibrosis. Indian J Gastroenterol.

[16] Crowley D, Cullen W, Laird E, Lambert JS, Mc HT, Murphy C, Van Hout MC (2017). Exploring patient characteristics and barriers to hepatitis C treatment in patients on opioid substitution treatment attending a community based fibro-scanning clinic. J Transl Int Med.

[17] Carew AM, Murphy N, Long J, Hunter K, Lyons S, Walsh C, Thornton L (2017). Incidence of hepatitis C among people who inject drugs in Ireland. Hepatol Med Policy.

[18] Cullen W, Bury G, Barry J, O'Kelly FD (2003). Hepatitis C infection among drug users attending general practice. Ir J Med Sci.

[19] Ryder N, Cullen W, Barry J, Bury G, Keenan E, Smyth BP (2009). Prevalence of problem alcohol use among patients attending primary care for methadone treatment. BMC Fam Pract.

[20] Field CA, Klimas J, Barry J, Bury G, Keenan E, Smyth BP, Cullen W (2013). Problem alcohol use among problem drug users in primary care: a qualitative study of what patients think about screening and treatment. BMC Fam Pract.

[21] Babor TF, Higgins-Biddle JC, Saunders JB, Monteiro MG http://apps.who.int/iris/bitstream/handle/10665/67205/WHO_MSD_MSB_01.6a.pdf?sequence=1.

[22] McGowan CE, Fried MW (2012). Barriers to hepatitis C treatment. Liver Int.

[23] Swan D, Long J, Carr O, Flanagan J, Irish H, Keating S, Keaveney M, Lambert J, McCormick PA, McKiernan S, Moloney J, Perry N, Cullen W (2010). Barriers to and facilitators of hepatitis C testing, management, and treatment among current and former injecting drug users: a qualitative exploration. AIDS Patient Care STDS.

[24] Wade AJ, Macdonald DM, Doyle JS, Gordon A, Roberts SK, Thompson AJ, Hellard ME (2015). The cascade of care for an Australian community-based hepatitis C treatment service. PLoS One.

[25] Zeremski M, Sylvester C, Talal AH (2016). Response to commentary on Zeremski et al. (2016): improvements in HCV-related knowledge among substance users on opioid agonist therapy after an educational intervention. J Addict Med.

[26] Cullen W, Stanley J, Langton D, Kelly Y, Bury G (2007). Management of hepatitis C among drug users attending general practice in Ireland: baseline data from the Dublin area hepatitis C in general practice initiative. Eur J Gen Pract.

[27] Cullen BL, Hutchinson SJ, Cameron SO, Anderson E, Ahmed S, Spence E, Mills PR, Mandeville R, Forrest E, Washington M, Wong R, Fox R, Goldberg DJ (2012). Identifying former injecting drug users infected with hepatitis C: an evaluation of a general practice-based case-finding intervention. J Public Health (Oxf).

[28] Klimas J, Marie Henihan A, McCombe G, Swan D, Anderson R, Bury G, Dunne C, Keenan E, Saunders J, Shorter GW, Smyth BP, Cullen W (2015). Psychosocial Interventions for Problem Alcohol Use in Primary Care Settings (PINTA): baseline feasibility data. J Dual Diagn.

[29] Henihan AM, McCombe G, Klimas J, Swan D, Leahy D, Anderson R, Bury G, Dunne CP, Keenan E, Lambert JS, Meagher D, O'Gorman C, O'Toole TP, Saunders J, Shorter GW, Smyth BP, Kaner E, Cullen W (2016). Feasibility of alcohol screening among patients receiving opioid treatment in primary care. BMC Fam Pract.

[30] Arain M, Campbell MJ, Cooper CL, Lancaster GA (2010). What is a pilot or feasibility study? A review of current practice and editorial policy. BMC Med Res Methodol.

[31] Klimas J, Anderson R, Bourke M, Bury G, Field CA, Kaner E, Keane R, Keenan E, Meagher D, Murphy B, O'Gorman CS, O'Toole TP, Saunders J, Smyth BP, Dunne C, Cullen W (2013). Psychosocial interventions for alcohol use among problem drug users: protocol for a feasibility study in primary care. JMIR Res Protoc.

[32] Campbell M, Fitzpatrick R, Haines A, Kinmonth AL, Sandercock P, Spiegelhalter D, Tyrer P (2000). Framework for design and evaluation of complex interventions to improve health. Br Med J.

[33] McCombe G, Henihan AM, Klimas J, Swan D, Leahy D, Anderson R, Bury G, Dunne C, Keenan E, Meagher D, O’Gorman C, O’Toole T, Saunders J, Smyth BP, Lambert JS, Kaner E, Cullen W (2016). Enhancing alcohol screening and brief intervention among people receiving opioid agonist treatment: qualitative study in primary care. Drugs Alcohol Today.

[34] Teddlie C, Yu F (2016). Mixed methods sampling. J Mix Methods Res.

[35] Braun V, Clarke V (2006). Using thematic analysis in psychology. Qual Res Psychol.

[36] Cullen W, Stanley J, Langton D, Kelly Y, Staines A, Bury G (2006). Hepatitis C infection among injecting drug users in general practice: a cluster randomised controlled trial of clinical guidelines' implementation. Br J Gen Pract.

